# Two-photon optogenetics-based assessment of neuronal connectivity in healthy and chronic hypoperfusion mice

**DOI:** 10.1117/1.NPh.11.3.035009

**Published:** 2024-09-28

**Authors:** Masaki Yoshioka, Manami Takahashi, Jeff Kershaw, Mariko Handa, Ayaka Takada, Hiroyuki Takuwa

**Affiliations:** a National Institutes for Quantum Science and Technology, Institute for Quantum Life Science, Quantum Neuromapping and Neuromodulation Team, Chiba, Japan; b Chiba University, Graduate School of Medicine, Department of Neurological Surgery, Chiba, Japan; c National Institutes for Quantum Science and Technology, Institute for Quantum Medical Science, Department of Molecular Imaging and Theranostics, Chiba, Japan; d Chiba University, Graduate School of Science, Department of Quantum Life Science, Chiba, Japan

**Keywords:** two-photon optogenetics, neuronal connectivity, calcium imaging, chronic cerebral hypoperfusion

## Abstract

**Significance:**

Two-photon optogenetics and simultaneous calcium imaging can be used to visualize the response of surrounding neurons with respect to the activity of an optically stimulated target neuron, providing a direct method to assess neuronal connectivity.

**Aim:**

We aim to develop a two-photon optogenetics-based method for evaluating neuronal connectivity, compare it to the existing indirect resting-state synchrony method, and investigate the application of the method to brain pathophysiology.

**Approach:**

C1V1-mScarlet was introduced into GCaMP6s-expressing transgenic mice with an adeno-associated virus. Optical stimulation of a single target neuron and simultaneous calcium imaging of the target and surrounding cells were performed. Neuronal connectivity was evaluated from the correlation between the fluorescence intensity of the target and surrounding cells.

**Results:**

The neuronal connectivity in the living brain was evaluated using two-photon optogenetics. However, resting-state synchrony was not always consistent with two-photon optogenetics-based connectivity. Comparison with neuronal synchrony measured during sensory stimulation suggested that the disagreement was due to external sensory input. Two-photon optogenetics-based connectivity significantly decreased in the common carotid artery occlusion model, whereas there was no significant change in the control group.

**Conclusions:**

We successfully developed a direct method to evaluate neuronal connectivity in the living brain using two-photon optogenetics. The technique was successful in detecting connectivity impairment in hypoperfusion model mice.

## 1 Introduction

Neurons within the brain flexibly control circuits by altering the synaptic connectivity between cells, enabling sophisticated information processing.^1^^,^^2^ Therefore, merely revealing the structure of the neural network (e.g., immunostaining on excised brain slice specimens) is insufficient for a comprehensive understanding of brain neural circuits. It is also necessary to understand the connectivity between neurons that form circuits. Studies of various brain disorders, such as dementia, stroke, and epilepsy, have reported decreases in the number of synapses and alterations in the connectivity between neurons.^3^^,^^4^ Understanding the influence of a disorder on synaptic connectivity is crucial for unraveling disease mechanisms and developing treatment strategies.

To evaluate the strength of connectivity between neurons, various methods have been employed, including patch clamping and two-photon microscopy-based calcium imaging of *ex vivo* brain slices and living brains.^5^^,^^6^ Previous studies have demonstrated a good correlation between synchronized neuronal activity obtained from *in vivo* calcium imaging and the connectivity between neurons obtained from *ex vivo* patch clamping.^5^ However, as the number of cells that can be simultaneously measured with electrodes is limited, it is difficult to assess complicated circuits with patch clamping. Two-photon microscopy-based calcium imaging is therefore the technique most commonly used to assess neuronal connectivity in the living brain. The neuronal connectivity *in vivo* is evaluated by calculating the correlation coefficient between neurons identified on a series of calcium-based images during the resting state.^7^ The correlation coefficient reflects the synchrony of neuronal activity between cells and serves as an indirect measure of neuronal connectivity. Unfortunately, when conducting measurements on living tissues, the occurrence of spontaneous sensory input and other factors may affect neuronal activity during resting-state measurements, potentially reducing the accuracy of the measurement of neuronal connectivity. A method to characterize the true connectivity between neurons in the living brain is therefore desirable.

Two-photon optogenetics uses laser light to induce neuronal activity in a single cell expressing channelrhodopsin. Moreover, red-shifted channelrhodopsin, which responds to light of a longer wavelength (530 nm) than that of conventional channelrhodopsin, allows optical stimulation and calcium imaging to be performed simultaneously.^8^^–^^10^ It is possible to induce neuronal activity in a specific target cell and observe the response in surrounding cells. This method enables direct visualization of the responsiveness of surrounding cells with respect to the activity of the target cell, providing an accurate assessment of the connectivity between neurons.^11^ Two-photon optogenetics has been used in previous studies to evaluate the connectivity between neurons in brain slice specimens, and the accuracy of the technique was validated with patch clamping.^12^^–^^15^

Although it has been shown that two-photon optogenetics is useful for assessing neuronal connectivity *ex vivo*, its utility for *in vivo* pathophysiological research remains to be determined. Earlier attempts to assess neuronal connectivity *in vivo* have used two-photon calcium imaging to demonstrate impaired connectivity in stroke and Alzheimer’s disease.^16^^–^^19^ However, two-photon calcium imaging alone is an indirect method of assessing connectivity. Two-photon optogenetics is a potential tool for directly assessing impaired neural connectivity resulting from brain disease.

In this study, we applied two-photon optogenetics to evaluate the connectivity between neurons in the living brain and compared the results with the resting-state neuronal synchrony measured with calcium imaging alone. The two methods were also compared with the connectivity measured during external stimulation. Finally, we investigated impairment in functional connectivity between neurons in a hypoperfusion mouse model exhibiting characteristics of neurofibril degeneration, such as behavioral learning and white matter lesions.

## 2 Materials and Methods

### 2.1 Animal Preparation

Male and female double-transgenic mice (20 to 30 g; aged >7 weeks) expressing GCaMP6s and CaMKIIα-Cre were used for *in vivo* two-photon calcium imaging in three types of experiments: (1) neuronal activity evoked by two-photon optogenetics, (2) resting-state imaging with no stimulation, and (3) neuronal activity evoked by air-puff stimulation of the whiskers. The mice were obtained by mating GCaMP6s transgenic mice (B6J.Cg-Gt(ROSA)26Sor<tm96(CAG-GCaMP6s)Hze>/MwarJ, No. 028866, The Jackson Laboratory, Sacramento, California, United States) and CaMKIIα-Cre transgenic mice (B6.Cg-Tg(Camk2a-cre)T29-1Stl/J, No. 005359, The Jackson Laboratory, Sacramento, California, United States). The animals were housed with food and water provided ad libitum in cages maintained at 25°C under a 12-h light/dark cycle. All experiments were conducted in accordance with institutional guidelines for the humane care and use of laboratory animals and were approved by the Institutional Committee for Animal Experimentation of the National Institutes for Quantum Science and Technology.

Three weeks before *in vivo* two-photon microscopic imaging, a chronic cranial window was attached to the animal’s skull above the left hemisphere of the brain. The animals were anesthetized with a mixture of air, oxygen, and isoflurane (3% to 5% for induction and 2% for surgery) via a facemask. Rectal temperature was monitored, and the animal was heated to maintain body temperature at 36°C to 37°C. The cranial window (4 to 5 mm in diameter) was positioned over the left somatosensory cortex following the “Seylaz-Tomita method,” so that its center was 1.8 mm caudal and 2.5 mm lateral to the bregma.^20^ During this surgery, adeno-associated virus (AAV) vectors carrying the C1V1 gene (pAAV-CamKIIa-C1V1(t/t)-mScarlet-KV2.1, No. 124650, $1\times 10^{13}\;\mathrm{vg}/\mathrm{mL}$, Addgene, Watertown, Massachusetts, United States) were injected into the barrel cortex using a glass needle. The soma-targeted C1V1 allowed specific single target neurons to be stimulated.^14^ Plastic bars 14 mm long were also attached to the front and rear ends of the skull so that the animal’s head could be stereotactically fixed under the microscope.

The surgical procedure used for unilateral common carotid artery occlusion (CCAO) has been described in previous studies.^21^^,^^22^ Briefly, a mixture of air, oxygen, and isoflurane anesthesia (3% to 5% for induction and 1.5% to 2% for surgery) was provided via a facemask. After a midline cervical incision was made, the left common carotid artery (ipsilateral to the cranial window) was isolated from the adjacent vagus nerve and double-ligated with 4–0 silk sutures.

### 2.2 Two-Photon Microscope Setting

A two-photon microscope (Thorlabs, Newton, New Jersey, United States) equipped with two independent lasers (Spark Lasers, Martillac, France) was prepared. One laser emitted 920 nm wavelength light, and the other emitted 1064 nm wavelength light. The 920 nm laser was used for calcium imaging of neuronal activity with a resonant mirror in its optical path. The 1064 nm laser, also with a resonant mirror installed in the optical pathway, was used for mScarlet imaging when confirming C1V1 expression. During the two-photon optical stimulation, a galvano mirror was introduced into the optical path of the 1064 nm laser to irradiate the cell body of the target neuron in an inward spiral pattern. The optical path of the 1064 nm laser was switched for imaging and optical stimulation using a mirror that was rotated by an electric motor. Different dichroic mirrors were used for imaging and optical stimulation. Two independent photomultiplier tubes, one for 460 nm imaging and the other for 532 nm imaging, were installed for signal detection. Before the optical stimulation experiment on each mouse, fluorescent microscope slides (Thorlabs) were used to verify that the 1064 nm laser accurately irradiated the target cell. The two-photon microscope was controlled by the ThorImage software (Thorlabs). The calcium images had a resolution of 512×512  pixels and field of view (FOV) of 600  μm×600  μm and were acquired at a frame rate of 15 Hz.

### 2.3 Selective Two-Photon Optogenetic Activation of Single Neurons

The experimental protocol used for measurements on awake mice has been reported previously.^23^^,^^24^ Briefly, the plastic bars on the animal’s head were screwed into a custom-made stereotactic apparatus. The animal was then placed on a styrofoam ball floating on a stream of air, which allowed the animal to exercise freely on the ball while its head was fixed to the apparatus.

We chose target cells and defined regions of interest (ROIs) for optical stimulation based on the presence of the red fluorescent protein mScarlet expressed in neurons. Layer 2/3 neurons 80 to 100  μm from the brain surface were targeted for optical stimulation. The laser pulse used for optical stimulation followed an inward “spiral” pattern as previous studies have demonstrated its effectiveness in inducing neuronal activity.^11^ The diameter of the optically stimulated area was set between 20 and 30  μm, which is large enough to completely cover the soma of the target neuron, and a stimulation duration of 30 to 50 ms was used. The laser intensity during stimulation was 88 mW, or 0.12 to $0.28\;\mathrm{mW}/{\mu m}^{2}$.

### 2.4 Magnetic Resonance Imaging of the Hypoperfusion Model Mice

Magnetic resonance imaging (MRI) measurements were performed 4 weeks after CCAO to evaluate reduction in cerebral blood flow. The experimental protocol was identical to that used in a previous study.^21^ Briefly, the MRI measurements were performed on mice under isoflurane anesthesia (3% to 5% for induction and 1.5% to 2% for surgery) using a 7-Tesla animal MRI system (Bruker, Billerica, Massachusetts, United States). Rectal temperature was continuously monitored and maintained at 36°C to 37°C using a heating pad. T2-weighted and CBF-ASL images were obtained using the same protocol as in our previous research.^21^ The CBF-ASL image was analyzed in NIH ImageJ.

### 2.5 Analytical Methods for Evaluating Connectivity between Neurons

The analysis of neuronal activity was performed in NIH ImageJ. We used “moco,” an optional function within ImageJ, to remove motion artifacts in serial images obtained during awake imaging. Moco is a template-based motion correction technique that can correct misalignment in the x−y direction but cannot correct motion artifacts in the *z* direction.^25^ Motion artifacts in the *z* direction were instead treated by removing frames where the fluorescence intensity of the image changed unnaturally (the number of frames removed was less than 10% of the total video length). ROIs were manually placed on the cell bodies of neurons in the image. To reduce the risk of missing neurons with poor baseline fluorescence, the maximum intensity was extracted with x−y−t projection. ROIs overlapping multiple neurons were excluded. The extracted signal was the average fluorescence intensity of all pixels belonging to the ROI. Connectivity between the target and surrounding neurons was assessed in three ways:

#### 2.5.1 Two-photon optogenetics-based neuronal connectivity

Percent changes in GCaMP6s fluorescence intensity (ΔF/F) induced by the optical stimulation were normalized with respect to the baseline (i.e., the average intensity in the 2 s prior to stimulation). ΔActivity was determined by averaging the ΔF/F data after optical stimulation ended (1 s; 15 frames). Pearson’s correlation analysis between the target cell and all other neurons was performed using the ΔActivity data from all trials (20 to 25 trials). The correlation coefficients obtained from the analysis represented the two-photon optogenetics-based neuronal connectivity.

#### 2.5.2 Resting-state neuronal connectivity

Percent changes in GCaMP6s fluorescence intensity (ΔF/F) during the resting state (400 s; 6000 frames) were normalized with respect to the baseline (taken as the 10-percentile intensity of the entire measurement period during the resting state). Pearson’s correlation analysis was conducted between the normalized ΔF/F data of the target cell and the data from all other neurons identified in the image.

#### 2.5.3 Neuronal connectivity during sensory stimulation

Similar to the analysis for two-photon optogenetics-based neuronal connectivity, percent changes in GCaMP6s fluorescence intensity (ΔF/F) induced by the sensory stimulation were normalized with respect to the baseline (the average intensity in the 2 s prior to stimulation). Pearson’s correlation analysis between the target cell and other neurons was performed on the ΔActivity data, defined as the averaged ΔF/F during the sensory stimulation period (4 s; 60 frames), for all trials (20 trials). In addition, the ΔF/F data beginning 6 s after sensory stimulation ended were extracted (∼13  s; 200 frames) for all 20 trials and combined (4000 frames) to assess neuronal synchrony after the mice had returned to a resting state. Pearson’s correlation analysis was performed on these data to assess the resting-state connectivity between the target and surrounding neurons post-sensory stimulation.

### 2.6 Statistical Analysis

Statistical comparisons were performed using a Friedman test. A significance level of 0.05 was selected.

## 3 Results

### 3.1 Two-Photon Optogenetics-Based Neuronal Connectivity

**Fig. 1:**
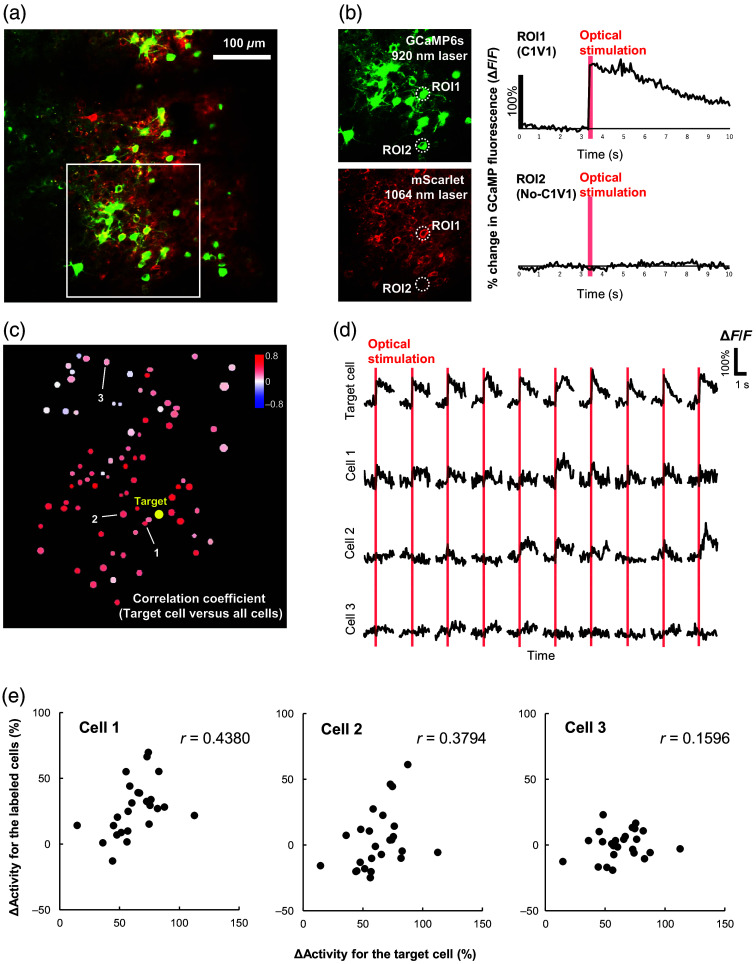
Two-photon optogenetics-based neuronal connectivity: (a) a typical two-photon imaging FOV of layer 2/3 in the somatosensory cortex. Neurons co-expressing GCaMP6s (green) and C1V1-mScarlet (red) can be observed with 920 and 1064 nm imaging, respectively. The area surrounded by the white square is shown in panel (b). (b) Neuronal activity induced using two-photon optical stimulation. A neuron expressing C1V1 (ROI 1) responds to optical stimulation, whereas a neuron without C1V1 (ROI 2) does not respond. (c) An example of a correlation coefficient map. The target neuron for optical stimulation is shown in yellow. The correlation coefficient of each cell with respect to the target cell is indicated according to the color scale in the top right of the image. (d) Percent change in GCaMP6s fluorescence (ΔF/F) of the target cell and the labeled cells (cells 1 to 3) induced after optical stimulation. The graph shows representative data from 10 of the 20 to 25 stimulation periods during the experiments. (e) Scatter plots of ΔActivity for the target cell and the labeled cells (cells 1 to 3). The *r*-value in the top right corresponds to the correlation coefficient calculated for the scattered data points.

We prepared mice co-expressing GCaMP6s and C1V1-mScarlet, which were excited by 920 and 1064 nm light, respectively [Fig. 1(a)]. C1V1-expressing cells were identified based on the presence of mScarlet, and target cells for optical stimulation were selected from these cells. Neurons expressing C1V1 (ROI 1) showed induced neuronal activity upon optical stimulation, whereas neurons without C1V1 (ROI 2) did not exhibit neuronal activity under optical stimulation [Fig. 1(b)]. An example of a correlation coefficient map is shown in Fig. 1(c). Each of the dots on the map represents a neuron observed within the 600  μm×600  μm FOV. There were usually 80 to 160 measurable neurons per FOV. Red dots indicate cells with high positive correlation coefficients with respect to the target cell (yellow), whereas dots indicate cells with low correlation coefficients with respect to the target cell. Blue dots indicate cells with negative correlation coefficients with respect to the target cell. In Fig. 1(d), ΔF/F increased after optical stimulation for neurons exhibiting high correlation with the target cell (cell 1). Conversely, neurons appearing white on the map showed little response after optical stimulation (cell 3). Scatter plots of the ΔActivity for the target cell versus ΔActivity for the labeled cells (cells 1 to 3) are presented in Fig. 1(e). The correlation coefficients calculated for these scatter plots are used to characterize the two-photon optogenetics-based connectivity between the target neuron and all surrounding neurons.

### 3.2 Comparison of Neuronal Connectivity Evaluated Using Two-Photon Optogenetics and Resting-State Synchrony

**Fig. 2:**
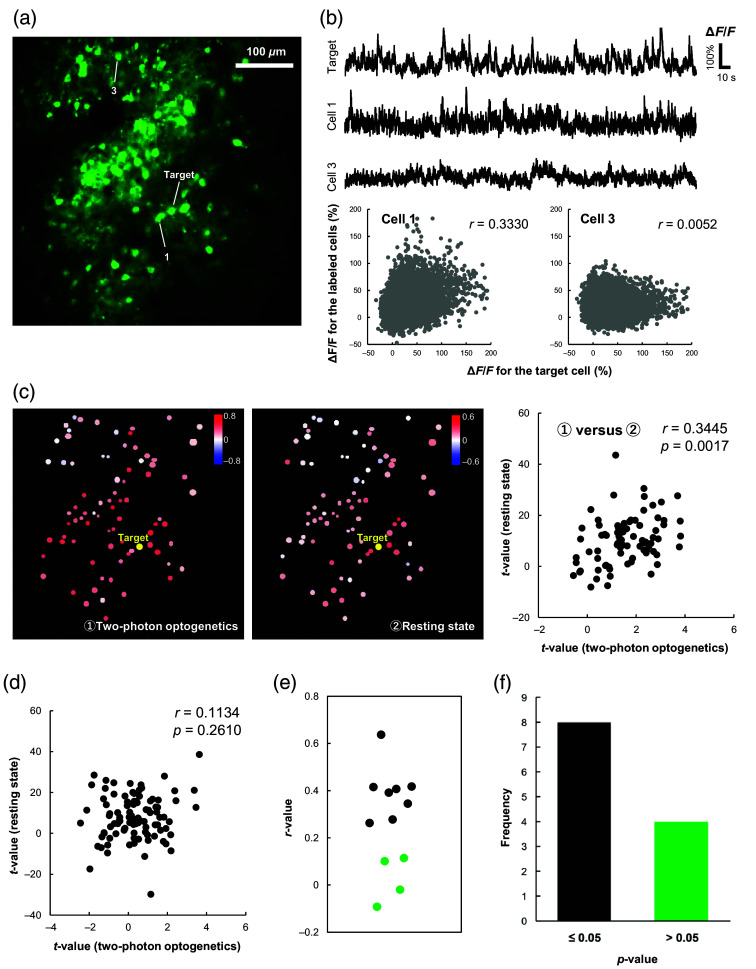
Comparison of neuronal connectivity evaluated using two-photon optogenetics and resting-state synchrony: (a) a representative FOV where the neuronal activity was assessed. Cells with green fluorescence are GCaMP6s-expressing neurons. (b) Percent changes in GCaMP6s fluorescence (ΔF/F) during the resting state (upper) and corresponding scatter plots of ΔF/F (lower) for the target and labeled cells in panel (a). Correlation coefficients characterizing the connectivity based on resting-state synchrony (*r*-values) are shown in the top right of the plots. (c) Comparison of correlation coefficient maps based on two-photon optogenetics (left) and resting-state synchrony (middle). The *t*-values calculated for the two methods are compared in a scatter plot (right). The correlation between these two sets of *t*-values is r=0.3445 (p<0.05) in this example. (d) An example where the two sets of *t*-values are poorly correlated (r=0.1134, p>0.05). Two target cells were selected for each mouse, making a total of 12 experiments. The distribution of the corresponding *r*-values is shown in panel (e), and a histogram of the *p*-values is in panel (f). The black dots in panel (e) correspond to cases where p<0.05 in panel (f). A similar relationship exists for the green dots.

A representative FOV where neuronal activity was assessed is shown in Fig. 2(a), where resting-state neural synchrony (6000 frames; 400 s) was calculated with respect to the target cell [Fig. 2(b)]. Neurons with high correlation coefficients in the resting state (cell 1) exhibit ΔF/F changes resembling those of the target cell, whereas neurons with low correlation coefficients (cell 3) exhibit less similarity with the target cell [Fig. 2(b)]. Figure 2(c) shows two-photon optogenetics and resting-state synchrony correlation coefficient maps obtained for the same FOV. The map constructed using the two-photon optogenetics method [Fig. 2(c), left] is visually similar to the map obtained with resting-state neural synchrony [Fig. 2(c), middle]. Due to the difference in the number of data points acquired for each method (25 trials versus 6000 frames), the correlation coefficients were converted from *r*-values to *t*-values, via $t=r(\sqrt{N-2}/\sqrt{1-r^{2}})$ so that the results of the two methods could be quantitatively compared. Note that the t-distributions corresponding to N=25 and N=6000 are very similar. The right part of Fig. 2(c) presents a scatter plot comparing the two sets of *t*-values. In this example, the two sets of t-values were positively correlated (r=0.3445, p<0.05), but there were cases where the two sets of *t*-values were poorly correlated [Fig. 2(d)]. Two target neurons were selected for each of the six mice, making a total of 12 data sets. For eight out of the 12 cases, the *r*-values were above 0.2630, and the corresponding *p*-values were less than 0.05 [Figs. 2(e) and 2(f)], indicating that the neuronal connectivity measured with the two-photon optogenetics-based method for these eight cases was highly consistent with the results of the resting-state synchrony method. On the other hand, there were four cases where the *r*-value was below 0.1135, meaning that the *p*-values were above 0.05 and the correlation between the two methods was poor. A possible reason for this is that living animals experience spontaneous sensory inputs. As these experimental data were measured from the barrel region of the somatosensory cortex, it is possible that sensory inputs may have affected the neural activity during rest. The evaluation of neuronal connectivity may become less accurate when there is an external sensory input that disturbs resting-state neuronal synchrony, leading to lower agreement between the two methods. To test this hypothesis, we evaluated the synchrony of neural activity while artificially introducing sensory inputs.

### 3.3 Evaluating Connectivity during External Sensory Stimulation: Comparison with Two-Photon Optogenetics-Based Connectivity

In this experiment, we introduced air-puff stimulation of the whiskers as a more potent sensory input. Because the measurements were conducted in the barrel region of the somatosensory cortex, it is expected that there will be increased neuronal activity in response to sensory input from the whiskers. If the target neurons respond to sensory stimulation, the neuronal activity induced in the target neurons and the surrounding neurons may synchronize. This synchrony poses a risk of overestimating the connectivity between neurons. We therefore analyzed measurements where the target neuron did not respond to sensory stimulation. Out of the 12 experiments performed in Sec. 3.2, there were eight cases where this situation occurred.

**Fig. 3:**
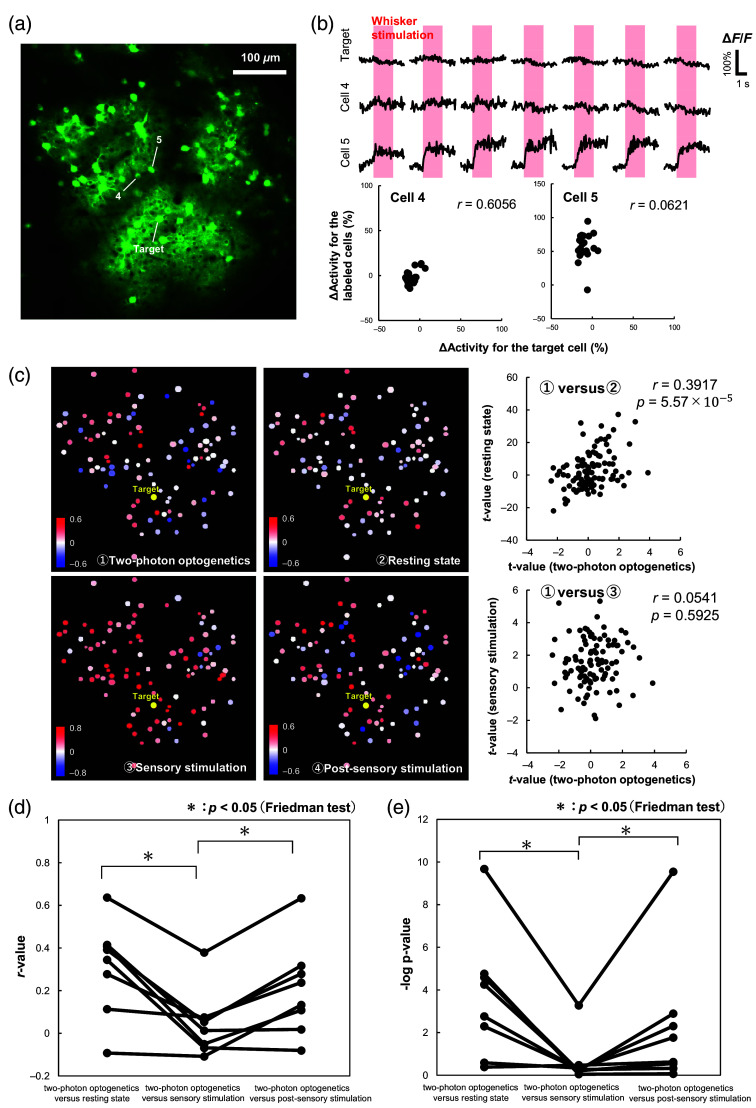
Comparison of two-photon optogenetics-based connectivity and synchrony during sensory stimulation: (a) another representative FOV different from that in Fig. 2 where the neuronal activity was assessed. (b) Percent change in GCaMP6s fluorescence (ΔF/F) during sensory stimulation (upper) and scatter plots of ΔActivity for the target and labeled cells (lower). The upper part shows representative data from seven out of 20 stimulation periods during the experiments. Correlation coefficients characterizing the connectivity based on synchrony during sensory stimulation (*r*-values) are shown in the top right of the plots. (c) On the left is a comparison of the correlation coefficient maps obtained with (1) two-photon optogenetics, (2) resting-state synchrony, (3) synchrony during sensory stimulation, and (4) resting-state synchrony after sensory stimulation. To the right of these maps are scatter plots comparing the *t*-values obtained for two-photon optogenetics with those obtained for resting-state synchrony and synchrony during sensory stimulation. (d) Comparison of the set of *r*-values obtained for two-photon optogenetics versus resting-state synchrony [same analysis as for Fig. 2(c)], the set of *r*-values obtained for two-photon optogenetics versus sensory stimulation [shown in panel (c), bottom right], and the set of *r*-values obtained for two-photon optogenetics versus resting-state after sensory stimulation. The results shown are for the eight experiments where the target neuron did not respond to the external sensory stimulation. The three sets of *r*-values were compared with the Friedman tests. (e) Comparison of the three sets of *p*-values accompanying the *r*-values. The three sets of *p*-values were also compared with Friedman tests.

In the representative FOV shown in Fig. 3(a), neuronal activity was measured during sensory input in addition to two-photon optogenetics and resting-state measurements. The upper part of Fig. 3(b) shows normalized ΔF/F changes for a target cell that did not respond to the sensory stimulation. A neuron with a large correlation coefficient (cell 4) exhibited similarities with the ΔF/F of the target neuron, and a neuron with a small correlation coefficient (cell 5) exhibited different ΔF/F changes from the target neuron. Correlation analysis was performed on the ΔActivity obtained by averaging ΔF/F during sensory stimulation [Fig. 3(b) lower]. Figure 3(c) presents correlation coefficient maps constructed using four methods: two-photon optogenetics, resting-state synchrony, sensory-stimulation synchrony, and resting-state synchrony after sensory stimulation. As in Sec. 3.2, the map constructed using the two-photon optogenetics-based method is visually similar to the map obtained with resting-state neural synchrony, and the two sets of *t*-values were significantly correlated [r=0.3917, p<0.05; Fig. 3(c), upper right]. However, the correlation coefficient map based on synchrony during sensory input is quite different from the map based on two-photon optogenetics, which is reflected by the two sets of *t*-values being poorly correlated [r=0.0541, p>0.05; Fig. 3(c), bottom right]. In addition, when the correlation coefficient maps calculated using resting-state synchrony after sensory stimulation were compared with those obtained with two-photon optogenetics, the two sets of *t*-values were positively correlated (r=0.3173, p<0.05; scatter plot not shown). Figures 3(d) and 3(e) show the results for the eight experiments where the target neuron did not respond to the sensory stimulation. In comparison to the two-photon optogenetics-based method, the correlation coefficients for resting-state neuronal synchrony had substantially higher significance (or lower *p*-values) than those measured to evaluate synchrony during sensory stimulation. The *r*-values (or *p*-values) for two-photon optogenetics versus resting state after sensory stimulation were also significantly higher (or lower) than those for two-photon optogenetics versus sensory stimulation [Figs. 3(d) and 3(e)]. A Friedman test applied to compare the three sets of *r*-values and *p*-values found a significant difference (p=0.0076 for *r*-values, p=0.0098 for *p*-values). The two-photon optogenetics-based connectivity was more consistent with resting-state synchrony and resting-state synchrony after sensory stimulation than the synchrony measured during sensory stimulation. This suggests that the evaluation of neuronal connectivity using resting-state synchrony is compromised when the resting-state neuronal activity is disturbed by spontaneous external stimulation.

Figures S1(a) and S1(b) in the Supplementary Material show the results for the four experiments where the target neuron responded to sensory stimulation. In three out of the four cases, the two sets of *r*-values and *p*-values exhibited the same trend. However, in one case, the *r*-value for two-photon optogenetics versus resting state was lower than that for two-photon optogenetics versus sensory stimulation.

### 3.4 Evaluation of Neuronal Connectivity in the Hypoperfusion Model Using the Two-Photon Optogenetics-Based Method

**Fig. 4:**
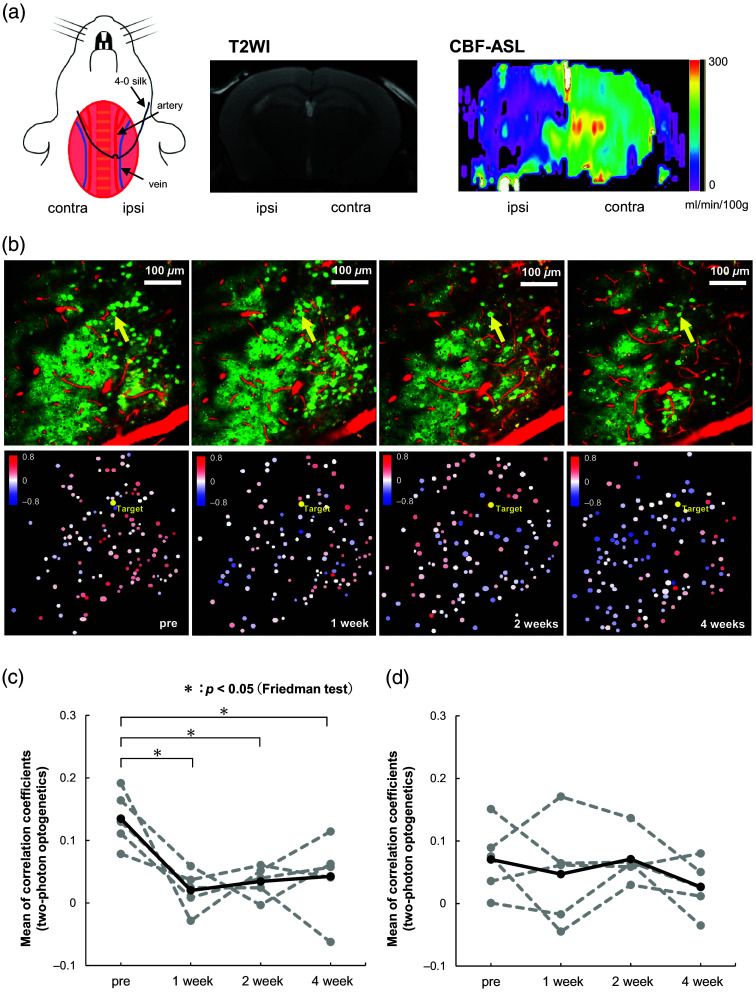
Evaluation of neuronal connectivity in the hypoperfusion model using the two-photon optogenetics-based method: (a) a mouse model of common carotid artery occlusion (CCAO). The left common carotid artery (ipsilateral to the cranial window) was separated from the jugular vein and the vagus nerve (not shown) to be ligated. In this model, there is no significant change in T2WI to indicate cerebral infarction despite reduced blood flow (CBF-ASL). (b) The upper row of images shows a representative FOV before (pre) and 1, 2, and 4 weeks after CCAO. The lower images show corresponding correlation coefficient maps. The target neurons are indicated by the yellow arrows in the upper images and by yellow dots in the lower maps. (c) Mean of the *r*-values for each animal (dashed lines) and the average over all five animals (solid line) before (pre) and 1, 2, and 4 weeks after CCAO. Differences between the means of the *r*-values at each of the four timepoints were evaluated with the Friedman test. (d) Mean of the *r*-values for each animal (dashed lines) and the average (solid line) for five control animals without CCAO measured at the same timepoints as for (c).

The chronic hypoperfusion mouse model used in this study involves the ligation of one side of the common carotid artery, resulting in an ∼30% sustained reduction in cerebral blood flow. However, no significant infarct lesions are formed [Fig. 4(a); Urushihata et al.^21^]. This model has been associated with pathological conditions such as white matter lesions, impairments in behavioral learning, and neuroinflammation.^26^^–^^28^ We have observed neurofibrillary impairments in the cerebral cortex and cerebral vascular dysfunction in this mouse model.^21^^,^^22^^,^^29^ Our experience with the CCAO model therefore made it an appropriate candidate for testing the two-photon optogenetics-based method in a pathological situation.

Two-photon optogenetics-based connectivity was measured by targeting the same neuron at four timepoints (i.e., just before CCAO, as well as at 1, 2, and 4 weeks after CCAO) in five animals. The left CCA was ligated during CCAO surgery [Fig. 4(a)] and all measurements were performed in the barrel cortex of the left hemisphere. As shown by the series of microscopic images in Fig. 4(b) (upper row), measurements were performed in the same FOV at the four timepoints. There was no decrease in neuronal activity and the frequency of neuronal firing for at least 1 week after CCAO surgery.^30^ For the target neurons, it was confirmed that the mScarlet fluorescence intensity, the location relative to the surrounding cells, the location relative to capillaries stained with sulforhodamine 101 (Adipogen Life Sciences, San Diego, California, United States), and the distance from the brain surface were not significantly altered over the 4 weeks of the experiments, which ensured that the target neurons for optical stimulation were identical at all timepoints. The visual impression from the maps is that hypoperfusion reduced the correlation coefficients over the imaged FOV [Fig. 4(b), lower row]. A Friedman test found that the average of the correlation coefficients for the five animals decreased significantly after CCAO [Fig. 4(c), p=0.026]. As a control experiment, we measured two-photon optogenetics-based connectivity at the same timepoints for five animals without CCAO and found no obvious change in the mean correlation coefficient [Fig. 4(d)].

The connectivity based on resting-state neural synchrony was also measured before and after CCAO in the same FOV. There was no significant change in the average of the correlation coefficients before and after CCAO [Fig. S2(a) in the Supplementary Material]. In addition, so as not to limit the analysis to the results for a single cell (i.e., the target neuron), a set of correlation coefficient maps was created using a different neuron as the target for each map in the set. The correlation coefficient was averaged over each map and then over the set to produce Fig. S2(b) in the Supplementary Material. The average correlation coefficients calculated in this way did not change significantly after CCAO.

## 4 Discussion

An important feature of neural circuits is the temporal flexibility in the connectivity between neurons.^1^^,^^2^ The plasticity within neural circuits is fundamental to realizing information processing in the brain. Functional impairment of neural circuits is associated with the pathogenesis of brain diseases. Previous studies on the pathophysiology of stroke with cerebral infarction, dementia, and epilepsy have reported decreases in synaptic density accompanying neural dysfunction.^3^^,^^4^ Assessment at the whole-brain level with MRI has also shown changes in the connectivity of neural circuits in psychiatric disorders.^31^^,^^32^ Accurate evaluation of the connectivity between individual neurons within the living brain is essential for unraveling the foundational mechanisms of the brain and understanding the pathophysiology of brain disorders. To date, only indirect methods have been available to assess the synchrony of neuronal activity. In contrast, the two-photon optogenetic technique activates a single neuron and observes the subsequent activity in surrounding cells, thus providing a direct method to assess the connectivity between neurons.^11^^–^^15^ It was expected that the two-photon optogenetics-based method could be used to characterize *in vivo* neuronal connectivity in brain pathophysiology research.

In this study, we applied the two-photon optogenetic method to evaluate neuronal connectivity in the living brain. Although the two-photon optogenetic technique has already been shown to be reliable *ex vivo*,^12^^–^^15^ some *in vivo* studies have also been performed.^11^^,^^33^^,^^34^ Chettih and Harvey^11^ used two-photon optogenetics to induce action potentials in target neurons and calcium imaging to measure the effects of stimulating the target neurons on neighboring neurons in awake mice. In contrast, the effects of action potentials in targeted excitatory neurons on neighboring neurons were visualized in this study using a more straightforward method of analysis [i.e., correlation analysis, e.g., Fig. 1(c)]. The relationship between the target and surrounding cells exhibits a range of responses from excitatory (positive correlations) to inhibitory (negative correlations). The results are consistent with what Finkelstein et al.^34^ have shown for tens of thousands of target and surrounding cell pairs using two-photon optogenetics.

We then compared the resting-state synchrony method for assessing connectivity to the two-photon optogenetics-based method. For eight out of 12 cases, the results demonstrated a high degree of similarity between the two methods (Fig. 2). However, the agreement was not so strong in four cases. Our hypothesis for this disagreement was that external factors, such as spontaneous sensory input, may have disturbed the resting-state neural activity. An experiment to test this hypothesis found that the significance of the connectivity between neurons decreased when external stimulation was applied (Fig. 3). These results suggest that two-photon optogenetics-based assessment of neuronal connectivity is less affected by external factors than the resting-state synchrony method. Note though that only a single neuron is stimulated using the two-photon optogenetic technique, so the network assessed by the method is limited to those neurons that are connected to the target neuron within the FOV. Thus, the two-photon optogenetics-based method has its limitations, and which method should be used depends on the purpose and situation to be assessed.

In previous studies, a middle cerebral artery occlusion mouse model exhibiting an infarct core demonstrated decreased neuronal connectivity when assessed with resting-state neuronal synchrony.^16^^,^^17^ In the CCAO mouse model used here, the mild reduction in cerebral blood flow does not lead to significant neuronal death.^21^ There were also no significant changes in the mean correlation coefficients after CCAO when assessed with the resting-state synchrony method [Figs. S2(a) and S2(b) in the Supplementary Material]. It is possible that the connectivity impairment due to CCAO was so mild that it was not measurable with resting-state neuronal synchrony. On the other hand, when employing the two-photon optogenetics-based method to evaluate neuronal connectivity, a significant decrease in the mean correlation coefficient was found over the 4 weeks after CCAO (Fig. 4). Furthermore, the mean correlation coefficient remained unchanged over the 4 weeks for mice without CCAO. These results demonstrate the impairment of neuronal connectivity in the chronic hypoperfusion model. The two-photon optogenetics-based method may be a more sensitive technique for assessing neuronal connectivity in the pathogenesis of brain diseases.

Baker et al.^14^ measured the propagation of neuronal activity to surrounding neurons using an electrophysiological method and revealed the connectivity of the neuronal circuit when a single neuron expressing soma-targeted channelrhodopsin was activated in brain slices. Izquierdo-Serra et al.^12^ also combined two-photon optogenetics and patch clamping to assess neuronal connectivity and showed that the strength of synaptic connections can be predicted from slight delays in action potentials. As the action potential delay they measured was only 20 to 60 ms, the effect of such a delay on the correlation analysis would be negligible at the temporal resolution of the calcium imaging used here (15 Hz). Assessment using two-photon optogenetics and simultaneous calcium imaging clearly reflects neuronal connectivity because changes in GCaMP fluorescence intensity are sensitive to changes in electrophysiological neural activity.^5^

The plasticity of synaptic connections is known to be strongly influenced by microglial activity. Removal of unneeded synapses in the normal brain is one of the functions of microglia.^35^^–^^37^ Activation of microglia by inflammatory responses can injure synaptic connections and cause impaired neural connectivity.^38^^,^^39^ Zhang et al.^28^ reported inflammatory microglial activation and subsequent white matter lesions after 1, 2, and 4 weeks of CCAO. Using diffusion tensor MRI, we have also reported neural fiber impairments within the brain parenchyma of mice after CCAO.^21^ The impairment in neuronal connectivity at 1, 2, and 4 weeks after CCAO revealed here with the two-photon optogenetics-based method is consistent with white matter lesions sustained after microglial activation. Therefore, the decrease in the mean correlation coefficient after CCAO observed in this study might correspond to neuronal fiber impairment caused by activated microglia.

In many previous optogenetics studies, the intensity of the laser light used to irradiate the target neuron *in vivo* varied from report to report.^8^^–^^11^^,^^40^ In our study, we performed single neuron stimulation with the laser intensity in the range of 22 to 110 mW and established that the optimum intensity for optical stimulation was 88 mW (0.12 to $0.28\;{\mathrm{mW}/\mu m}^{2}$) (Fig. S3 in the Supplementary Material). Such a laser intensity is not high enough to affect brain tissue temperature.^40^ Moreover, it has been suggested that crosstalk between imaging and optical stimulation may induce unintended activation of C1V1-expressing cells.^33^ We chose the calcium indicator GCaMP6s and the red-shifted channelrhodopsin C1V1-mScarlet to minimize excitation spectrum overlap. However, the intensity of the imaging laser must be kept at a low level because C1V1 has a slight green-light absorption tail. As Yang et al.^10^ reported that effects on C1V1 only occur at imaging laser intensities above 90 mW, we performed calcium imaging at laser intensities below 80 mW. It should also be noted here that an experiment was performed to test whether connectivity is affected by repeated application of the optical stimulation. After 25 repetitions of the stimulation, correlation analysis showed that neuronal connectivity was not altered (Fig. S4 in the Supplementary Material).

While assessing neuronal connectivity, some of the surrounding cells were found to be negatively correlated with the target neuron. Cells exhibiting negative correlation coefficients with respect to the target neuron (blue dots in the correlation coefficient maps) followed one of two patterns: (i) the cell had no clear change in ΔF/F when there was a substantial change in ΔF/F for the target neuron, or (ii) the cell had a negative ΔF/F relative to the positive ΔF/F of the target neuron. These patterns suggest that the negatively correlated cells were receiving inhibitory signals from the target neuron. However, as the only cells that can be visualized with calcium imaging are excitatory neurons expressing CaMKII, the inhibitory-like response may be transmitted via inhibitory neurons that are not present in the observed FOV. The comparison of the average correlation coefficients after CCAO in this study includes cases with an increased proportion of negatively correlated neurons [e.g., Fig. 4(b), 4 weeks]. The increased fraction of negatively correlated neurons could be caused by an imbalance between excitatory and inhibitory synaptic connectivity. It is reported that stroke causes a change in the excitatory and inhibitory balance as a phenotype of neuronal connectivity impairment.^3^^,^^41^ Kudo et al.^42^ showed that activated microglia destroy perineuronal nets and cause interneuron dysfunction in a mouse model of dementia. Therefore, the excitatory and inhibitory balance might also be affected in the CCAO mouse model, where microglia are activated and neuroinflammation occurs.^28^

In this study, Pearson’s correlation analysis was employed to assess synchrony during the resting state. We also considered the possibility of synchronized neural activity with some delay. The results of the shifted cross-correlation analysis showed that the synchrony of neural activity was largest when there was no time difference (Fig. S5 in the Supplementary Material). Therefore, we concluded that a correlation analysis considering time differences was unnecessary.

There are some limitations to the assessment of neuronal connectivity using two-photon optogenetics and simultaneous calcium imaging. It is possible that the activity of some neurons within the FOV is not detected because their calcium level changes are subthreshold. Furthermore, two neurons characterized to have robust connectivity with the two-photon optogenetics-based method do not necessarily have direct synaptic connections. Calcium imaging cannot visualize individual synapses *in vivo*, so the presence of synaptic connections cannot be confirmed in the same way as it can be for *ex vivo* brain slices. Therefore, the neuronal connectivity evaluated in this study may reflect connections through multiple neurons as well as direct one-to-one connections.

It should also be noted that images of the living brain taken with a two-photon microscope have a limited FOV and a lack of spatial resolution in the *z*-direction, which means that only a fraction of the cells that constitute a neural circuit can be observed. The number of neurons that are affected by the activity of the target cell varies in different FOVs and animals, leading to the variation in the mean correlation coefficients across animals in Figs. 4(c) and 4(d). Even though the FOV and the target neuron for optical stimulation were identical, we were unable to observe the exact same set of surrounding neurons at all timepoints, meaning that the observed neural circuit is not really “constant.” For that reason, some analysis was also performed using only neurons that were clearly observable at all timepoints (Fig. S6 in the Supplementary Material). The results are consistent with those in Sec. 3.4.

In conclusion, we have developed a method to evaluate neuronal connectivity *in vivo* and applied it to assess brain disorder pathophysiology. The results demonstrated that two-photon optogenetics has promise as a direct method for evaluating neuronal connectivity in the living brain and hypoperfusion model mice. We anticipate future application of the method to dementia and psychiatric disorders.

## Supplementary Material



## Data Availability

Code and data are available from the corresponding author upon reasonable request.
