# Supplementary material for: Two-photon optogenetics-based assessment of neuronal connectivity in healthy and chronic hypoperfusion mice
Source: Neurophotonics. 2024 Sep 28;11(3):035009. doi: 10.1117/1.NPh.11.3.035009 (PMC11436461; doi:10.1117/1.NPh.11.3.035009)
Supplement: Supplementary file 1 [file NPh_011_035009_SD001.pdf]

Supplemental Materials

In Addition to Peason's correlation analysis, the analysis was modified to calculate the cross-correlation coefficient after shifting one of the data sets by a time τ.

$$\text{Shifted Cross-Correlation} = \frac{S_{X(t)Y(t+\tau)}}{S_{X(t)}S_{Y(t+\tau)}} = \frac{\sum_{t=1}^{T}(X(t)-\bar{X})(Y(t+\tau)-\bar{Y})}{\sqrt{\sum_{t=1}^{T}(X(t)-\bar{X})^2}\sqrt{\sum_{t=1}^{T}(Y(t+\tau)-\bar{Y})^2}},$$

$$\bar{X} = \frac{1}{T}\sum_{t=1}^{T} X(t), \bar{Y} = \frac{1}{T}\sum_{t=1}^{T} Y(t+\tau)$$

Shifted Cross-Correlation was performed to evaluate the connectivity when there is a possible time delay in the transmission of neural activity. The Shifted Cross-Correlation value will be maximal at τ= 0 if there is no significant delay between excitatory neurons (Fig. S5).

Fig. S1

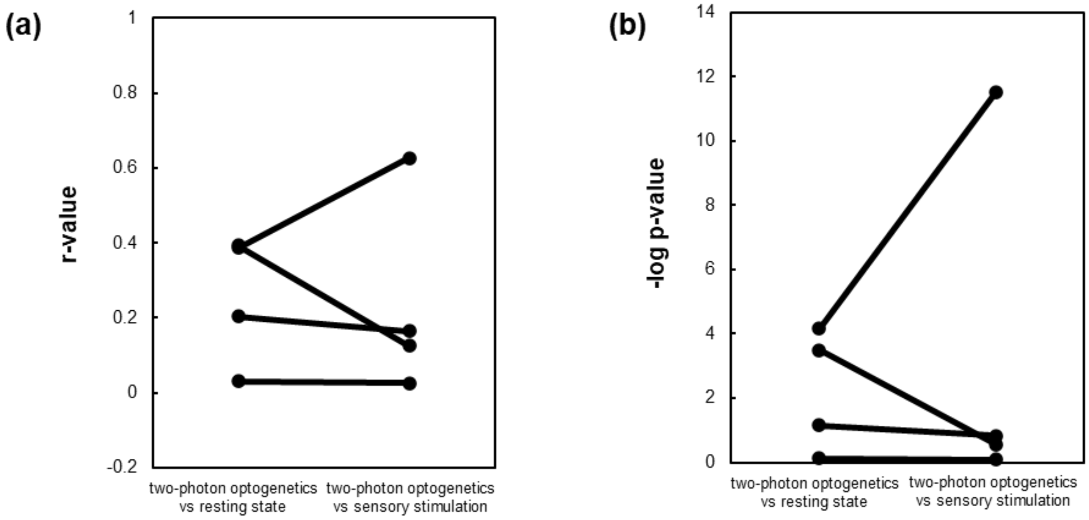

Fig. S1 Comparison of two-photon optogenetics-based connectivity and synchrony during sensory stimulation for the 4 experiments where the target neuron responded to the external sensory stimulation: (a) Comparison of the set of r-values obtained for two-photon optogenetics vs resting state (same analysis as for Fig. 2c), and the set of r-values obtained for two-photon optogenetics

vs sensory stimulation (same analysis as for Fig. 3c). (b) Comparison of the two sets of p-values accompanying the r-values.

Fig. S2

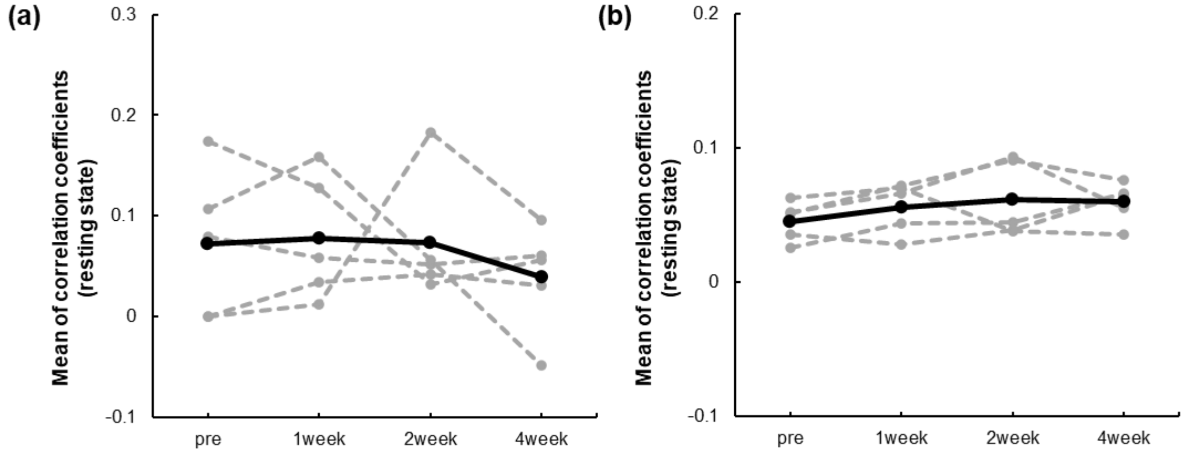

Fig. S2 Evaluation of neuronal connectivity in the hypoperfusion model using resting-state synchrony: (a) Mean of the correlation coefficients for each animal (dashed lines) and the average over all five animals (solid line) before (pre) and 1, 2, and 4 weeks after CCAO. Correlation coefficients were calculated in the same way as for Fig. 2b. (b) Mean of the correlation coefficients measured for each animal during the resting state using each neuron as the target and then averaging over all of the resulting maps (dashed lines). The solid line is the average over all five animals.

Fig. S3

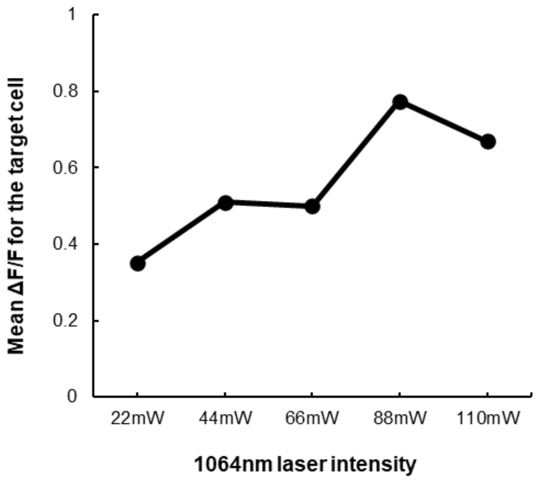

Fig. S3 Optimization of laser intensity for optical stimulation: Mean of the percent change in GCaMP6s fluorescence (ΔF/F) of the target cell is plotted against laser intensity (22, 44, 66, 88, 110 mW). The experiment was repeated eight times for six different neurons selected as the target for optical stimulation.

Fig. S4

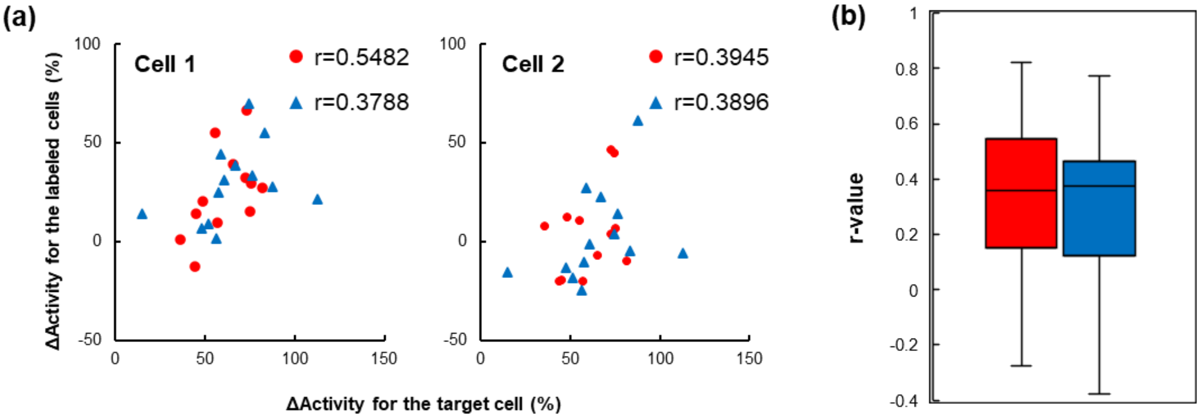

Fig. S4 Change in correlation coefficients between the target and surrounding neurons depending on the number of trials: (a) Scatter plots show ΔActivity for the target cell and the labeled cells shown in Fig. 1e, where red circles indicate results from the first half of the experiment (13 trials),

and blue triangles from the second half (12 trials) of the experiment. Correlation coefficients for the first and second halves are shown in the upper right corner of the scatterplot. (b) A box-and-whisker plot shows the distribution of correlation coefficients for all of the neurons surrounding the target neuron measured in the same animal as for Fig. 1c-e. Correlation coefficients calculated from the first half of the experiment are shown in red and the second half in blue. A t-test showed that there was no significant difference between these two sets of correlation coefficients (p=0.170).

Fig. S5

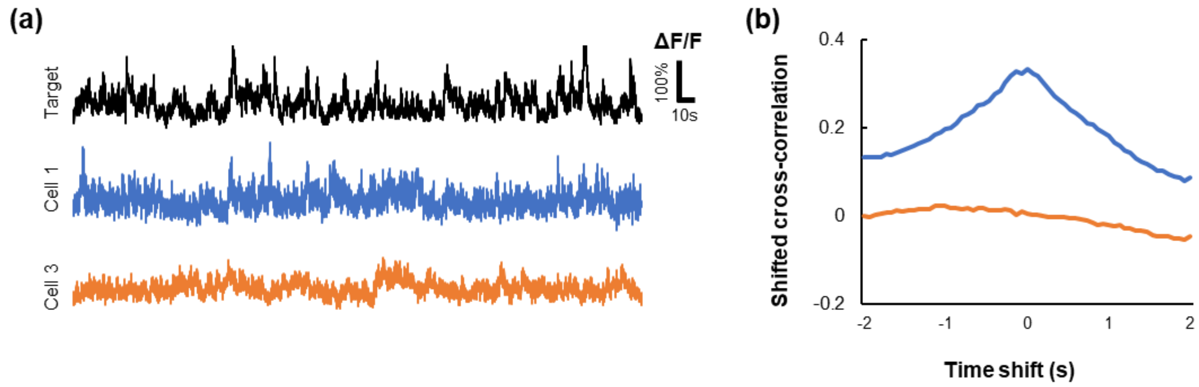

Fig. S5 Shifted cross-correlation analysis during the resting state: (a) Percent changes in GCaMP6s fluorescence (ΔF/F) during the resting-state (same data as Fig. 2b). (b) Shifted cross-correlation calculated from the normalized resting-state ΔF/F data. The shifted cross-correlation between the target neuron and cell 1 (blue line) was largest when there was no time difference, while that between the target neuron and cell 2 (orange line) was near zero regardless of the time shift.

Fig. S6

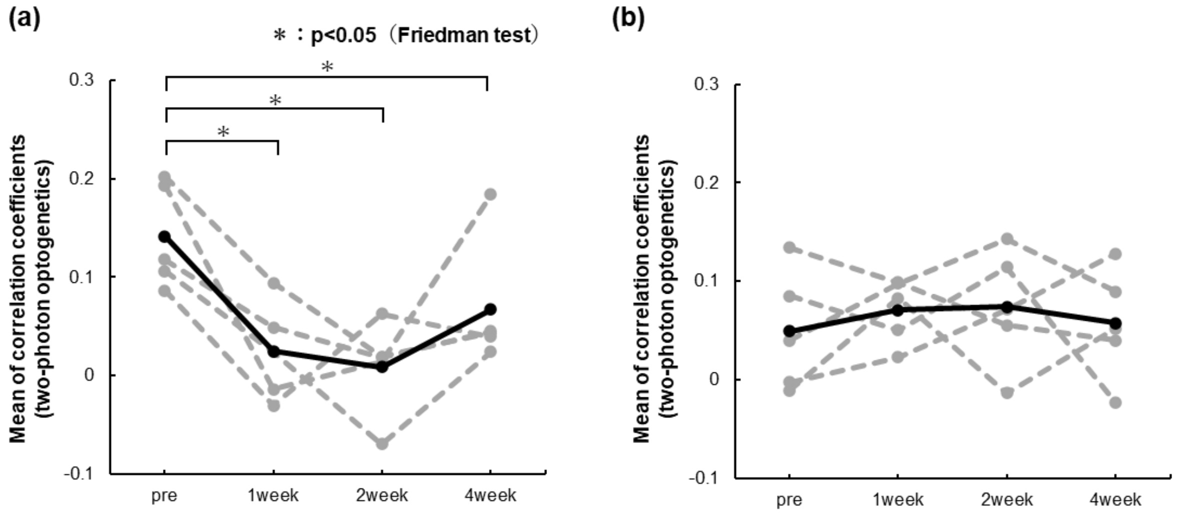

Fig. S6 Change in correlation coefficient for hypoperfusion model mice when the analysis was confined to neurons that were clearly visible at all timepoints: (a) Mean of the r-values for each animal (dashed lines) and the average over all five animals (solid line) before (pre) and 1, 2, and 4 weeks after CCAO. (b) Mean of the r-values for each animal (dashed lines) and the average (solid line) for five control animals without CCAO measured at the same timepoints as for Fig. S6a.
